# A Critical Review of the Science on “Highly” Processed Food Intake and Body Weight

**DOI:** 10.1016/j.advnut.2026.100683

**Published:** 2026-06-17

**Authors:** Giorgia Rutigliani, Joseph V Balagtas, Richard D Mattes

**Affiliations:** 1 Department of Nutrition Science, Purdue University, West Lafayette, IN, United States; 2 Department of Agricultural Economics, Purdue University, West Lafayette, IN, United States

**Keywords:** human, obesity, food intake, ultraprocessed, diet quality, food insecurity, dietary guidance, health risk

## Abstract

There is increasing consideration of food processing and formulation in setting dietary guidance at the state, federal, and global levels to manage obesity and other health conditions. However, the supporting evidence base is highly controversial. This stems, in part, from the lack of a widely accepted definition of highly or ultraprocessed foods and beverages (HPFB) and evidence that published definitions hold different predictive health risks. The preponderance of evidence linking food processing and formulation practices to health outcomes is drawn from observational studies. This literature generally suggests that HPFB intake is associated with a higher risk of obesity. However, a critical assessment indicates that the associations are weak and inconsistent. More recently, a number of randomized controlled trials have been published based on educational, community-based dietary and metabolic ward feeding interventions. Metabolic ward trials involved small samples, short-term assessment, and ecologically questionable comparisons. They reported no changes in appetite, and only transient shifts in food intake. The community-based trials generally have high attrition rates and use no objective measure of dietary compliance with the intervention. They reveal that the implementation of interventions to moderate HPFB intake is often not achieved. No community-based trial has observed weight gain on the high HPFB treatment arm, and only half report a significant group weight differential. Among all trials, the intake of HPFB has led to increased, no change, and decreased body weight. To date, they do not adequately support a causal relationship between HPFB intake and obesity risk. Given the tangible likely adverse effects of eliminating HPFB from the diet, such as increased food-borne illness, greater food waste, as well as decreased diet quality, increased food cost, and reduced convenience, especially for segments of the population least able to tolerate such changes, dietary guidance based solely on the HPFB concept is, presently, not in the public’s best interest.

**Statement of significance**

The epidemiological, randomized controlled trial, and mechanistic data linking consumption of highly processed foods and beverages with obesity risk are weak and inconsistent. Given the likely adverse consequences, recommendations for large-scale removal of such items from the diet based solely on their processing category may not be in the best interests of the population.

## Introduction

The health consequences of consuming diets containing highly processed foods and beverages (HPFB), how ever they are defined, remain controversial [[1], [2], [3]]. This stems, in part, from a lack of agreement on the best approach to classify foods on this property, or set of properties, and a heavy reliance on associations derived from epidemiological studies [[4], [5], [6], [7], [8], [9]]. The evidence base derived from randomized controlled trials is growing but still limited, and there are even less empirical data on the multiple proposed mechanisms of action. The present review seeks to bring focus on the evidence base to date to permit objective assessment of the current state of knowledge and where future research efforts may be best directed. We use the abbreviation HPFB when discussing the topic generally and ultraprocessed food (UPF) and minimally processed food (MPF) when referring to foods classified according to the NOVA system. This article is a narrative synthesis of the literature, based on targeted searches of PubMed, Google Scholar, and the Cochrane Library, along with reference screening of key publications. This approach was adopted because it was not deemed feasible to cover and synthesize the different forms of science—observational, randomized controlled trial (RCT), and mechanistic trials addressing many and varied outcomes (e.g., appetite, intake, and body weight) through other, more systematic analytic methods.

## Definition of Processed Foods

The lack of a universally agreed-upon definition of UPFs and beverages (UPFB) is a fundamental obstacle to both evaluation of current knowledge and identification of future research needs. This point was clearly highlighted by a study that classified foods according to 4 different definitions and examined the associations with various cardiometabolic health outcomes using a common database, the PREvención con DIeta MEDiterránea-Plus (PREDIMED-PLUS - a large-scale, long-term clinical trial conducted in Spain ) Cohort of 7447 individuals [10]. The definitions used were based on: *1*) the NOVA classification system identifying foods that are branded, commercial formulations made from cheap ingredients extracted or derived from whole foods and combined with additives [2] emphasizing the nature, extent, and purpose of food processing; *2*) the International Agency for Research on Cancer (IARC) of the WHO focusing on the level of processing; *3*) the International Food Information Council (IFIC) concentrating on the complexity of processing and formulation; and *4*) the University of North Carolina system highlighting physicochemical changes in foods. The analysis revealed that there was very low concordance between the classification systems. Fewer than one-third of individuals were assigned to the same intake quintile by NOVA and IARC (28%), NOVA and University of North Carolina (UNC) (30%), or NOVA and IFIC (32%). Even in the best-aligned pair (IFIC compared with UNC), concordance reached only 48.6%. Agreement at the extremes, the lowest (Q1) and highest (Q5) quintiles, where health effects are typically evaluated, was often below 10%–15%. Thus, the individuals identified as “high consumers” of highly processed foods differed substantially depending on the classification scheme. This discordance translated directly into divergent health inferences (Table 1). For example, a positive association was noted for BMI with the NOVA system, but not with the others; classification based on the IARC system led to an association with glycated hemoglobin, unlike any of the others, and using the UNC definition resulted in a positive association with blood pressure, whereas this was not observed with the other systems. Such discrepant findings highlight the importance of defining HPFBs and how the lack of a clear definition complicates the study of the consequences of their consumption. Indeed, the 2025 Dietary Guidelines Advisory Committee, which was charged with assessing this literature, did not draw any firm conclusions largely because of the lack of a clear definition. Their dilemma was compounded by evidence that was not even directionally consistent, as exemplified by whole grains, where, based on “strong evidence,” they recommended increased intake, yet many are classified as ultraprocessed [[11], [12], [13]].

**TABLE 1:** Observed associations between HPFB intake and health outcomes by classification system

	NOVA	IFIC	UNC	IARC
BMI	+	NS	NS	NS
Total cholesterol	NS	+	+	+
HbA1c	NS	NS	NS	+
Blood pressure	NS	NS	+	NS

Summary of outcomes showing a positive association with HPFB intake differs depending on the classification scheme.

“+” indicates a positive association between HPFB intake (as defined by the specified classification system) and the listed outcome in the referenced analysis. NS indicates no positive association observed for that outcome under that classification scheme.

Abbreviations: HbA1c, glycated hemoglobin; HPFB, highly processed food and beverage; IARC, International Agency for Research on Cancer system; IFIC, International Food Information Council system; NOVA, NOVA classification; UNC, University of North Carolina system.

The importance of further efforts to derive a widely agreed-upon definition is increasingly recognized. On 23 July, 2025, the Secretaries of United States Department of Health and Human Services and USDA issued a joint request for information to develop a uniform definition of UPFs [14]. Similarly, the WHO is populating an expert committee to provide guidance on a definition, as are others [15]. In this void, multiple states are considering legislation defining UPFs, and multiple definitions have been proposed [[16], [17], [18]]. None of which accounts for the growing recognition that there are food groups, such as selected grain products (e.g., breads and cereals), sweet and savory snacks, and yogurts, deemed highly processed by some classification systems, but have neutral or beneficial effects on cardiometabolic indices [12,13] and diabetes risk [19]. A categorization system for HPFBs that permits measurement of a causal relationship between intake and adverse effects is a necessary condition for policies, interventions, and innovations that could reduce risks. If there is a categorization system that truly captures a causal relationship between intake and adverse effects of consumption of HPFB, efforts to thoughtfully and effectively displace them from the diet would be desirable. However, if there is no causal relationship or one not captured by the definition, the potential adverse consequences of removing HPFB from the diet could be profound. Diet quality may be compromised by eliminating enriched and fortified foods [[20], [21], [22]]. Elimination of foods with preservatives would likely result in higher food-borne illness [23], greater food waste [24], and higher food costs [25,26], the latter being disproportionately problematic for people with food insecurity, living in rural areas and/or with limited access to refrigeration. The reduction in convenience foods would also complicate the lives of people living in single-parent families who rely on such items to manage the elevated time-based stresses on their lives [27]. HPFB also actually aids meeting nutritional goals of some segments of the population, like vegans and vegetarians [28,29]. A recent content analysis of 106 dietary guidance documents from around the world reported that advice about consuming processed foods is present in 86% (91 of 106) of the cases. Thus, it is imperative that the issue of a definition be resolved promptly.

## Observational Evidence

Despite the lack of a clear definition of HPFB, there has been an abundance of epidemiological studies exploring the associations between their consumption and a wide array of health outcomes [7]. This large and growing literature generally indicates that greater consumption of HPFB is associated with adverse health outcomes. Indeed, the volume and consistency of findings have prompted calls to accept this evidence base as sufficient to guide nutrition policy [2]. Historic examples where such an approach did not serve the public interest should temper such a suggestion. Recommendations, based primarily on observational evidence, to increase the dietary intake of beta carotene and retinol palmitate to manage cardiovascular disease were ill-advised as higher intake was associated with increased risk of lung cancer and cardiovascular mortality in females [30,31]. Furthermore, the low-fat guidelines introduced in the late 1970s prompted a massive shift toward reduced-fat foods to curb the rise in heart disease and obesity that was of no particular value [[32], [33], [34], [35], [36], [37]]. Concern about acceptance of, and taking action on, the existing observational evidence is also prudent given a number of outstanding issues with that evidence base. First, although most meta-analyses exploring the association between consumption of HPFB and multiple outcomes show a significant positive relationship, the effect sizes vary substantially by outcome and exposure scaling. Here, we focus on obesity, and when modeled per incremental increase in intake, effect sizes are often small, with relative risk or odds ratios for obesity in the range of 1.02–1.10 [38,39], although somewhat larger estimates, approaching ∼1.5, have been reported [7]. This level of risk, in the case of obesity, is less than or no greater than that documented for education level [40], economic status [41], sleep duration [42], anxiety [43], physical activity [44], television viewing [45], and numerous other proposed drivers of weight gain. Thus, although an association is present, arguably, it warrants no more action than many other well-established modifiable behavioral risk factors.

**FIGURE 1:**
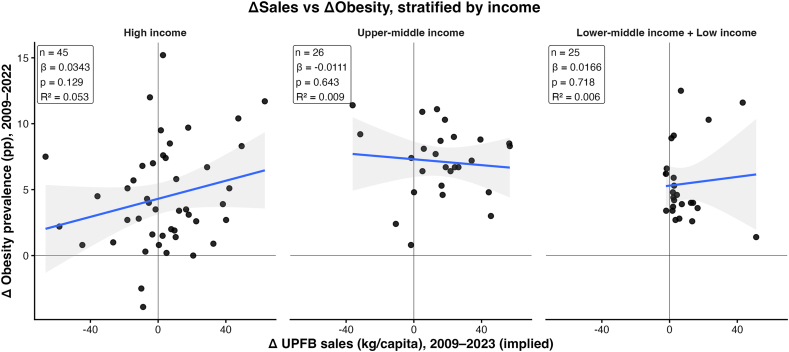
Change in national obesity prevalence vs. implied change in UPFB sales, stratified by income. To construct Figure 1, source of data for the absolute change in sales (kg/capita) were obtained from Baker et al. [49]. The data were digitized using WebPlotDigitizer [50] extracting per-capita UPFB sales in 2023 (kg/capita) and the reported growth in sales from 2009 to 2023 (%). Absolute change in sales over 2009–2023 was then derived in kg/capita by converting the reported growth to an implied 2009 baseline ($S_{2009}=S_{2023}/\left(1+g\right))$and computing $\Delta Sales=S_{2023}-S_{2009}$. These estimates were linked to national obesity prevalence from the WHO [51] for 2009 and 2022 to compute change in obesity (percentage points). We regressed the change in HPFB sales between years 2009 and 2023 (ΔSales) on the change in obesity rate from 2009 to 2022 *(*ΔObesity) for different WHO-defined national income categories (low/lower-middle, upper-middle, and high income. Only 2 low-income countries were included in the source dataset. HPFB, highly processed food and beverage; UPFB, ultraprocessed food and beverage.

Second, the evidence linking changes in consumption of HPFB to adverse health outcomes is not consistent with observed empirical trends. In the United States, obesity prevalence increased markedly between 1980 and 2016, from ∼12% to almost 35% of the population [46]. However, during that timeframe, the percent of consumed foods deemed HPFB oscillated between 3% lower and 4% higher relative to a 1988 intake estimate [47]. High, but stable, intake levels are observed in all segments of the United States population [48]. An inconsistency is also apparent for international comparisons of sales compared with obesity rates. In a recently published article reviewing the commercial determinants of UPFB intake, data were presented relating national sales growth of UPFB between 2009 and 2023 (%) to per-capita UPFB sales in 2023 (kg/capita) in 97 countries [49]. To our knowledge, this is the largest such analysis and covers a longer time span than other published work. It was noted that the transition to UPFB was intended to maximize industry profits, but there was no assessment of the health consequences of the documented changes in UPFB sales. To evaluate the public health relevance of these patterns, we linked UPFB sales estimates to national obesity prevalence values from the WHO (Figure 1). We observed no statistically significant associations in any income stratum. Moreover, sales growth accounted for only 0.6%, 0.9%, and 5.3% of the cross-country variation in obesity prevalence in the low/low-middle, upper-middle, and high-income countries, respectively.

Our findings are in line with prior similar analyses. In an assessment of 90 countries, Eaton et al. [52] also observed no significant association between UPF sales and obesity prevalence. A 1 SD increase of UPF sales was associated with 0.11 and 0.27 increments in BMI in males and females, respectively, over the period from 2005 to 2016. Assuming 1 BMI unit corresponds to about 6–8 pounds (using 7 as a mean), this translates to an increase of body weight of 0.064–0.158 pounds per year. Thus, the statistical significance may not be matched by practical importance. Small and inconsistent findings were provided in another report involving 80 countries assessed between 2002 and 2016 [53]. There, a SD increase in sales (40 kg/capita, relative to year 2002) of UPF, was associated with a statistically significant, but small 0.316 increment of BMI (∼0.157 pounds/y) in males, but was negative (–0.004) and not significant in females. Our null findings should be interpreted cautiously. The exposure is a commercial sales metric (foods and beverages combined), not measured dietary intake, and is subject to potential substantial error arising from food waste, informal markets, trade flows, and the dominance of beverages in mass-based units. Moreover, the 2009 sales baseline is inferred from a reported growth metric whose precise definition (cumulative percentage change compared with compound annual growth) is not specified in the source article and may differ from our assumptions. Such sources of error would be expected to attenuate any true association. The analysis is also ecological and therefore vulnerable to structural confounding by income, urbanization, retail development, and other macroeconomic forces that independently influence diet and obesity. Nonetheless, this exercise illustrates that the cross-national sales patterns presented by Baker et al. [49] do not, as plotted, translate into a detectable signal in contemporaneous obesity trends. As such, these commercial metrics, in their current form, do not provide a robust quantitative basis for causal inference or for guiding dietary policy. It should also be noted that rising obesity rates have been documented long before modern food processing techniques were implemented [[54], [55], [56]]. Thus, HPFB exposure is neither necessary nor sufficient to account for obesity trends.

Third, as noted above, the associations between UPFB intake and various health outcomes change with the definition of UPFB. Thus, at present, without an agreed-upon definition of UPFB, it is not possible to identify and quantify the consequences of consuming such foods and beverages. This would hamper any attempt to act upon the concept.

Fourth, there is a long-standing, robust literature linking added sugar, salt, and saturated fat intake with adverse health outcomes [[57], [58], [59], [60]]. These food components are commonly present in HPBF [14], raising concern about residual confounding. That is, reported associations between UPBF may be more attributable to these ingredients than the cited nature or degree of processing.

Fifth, there are subgroups of the population that benefit from UPBF consumption and could be harmed by their removal from the food supply. For example, among vegans, the ability to meet protein needs is lower on minimally processed diets and higher on diets with UPFs, both in a dose–response manner [29]. Foods that are enriched and fortified are largely considered HPFB. They do tend to have higher concentrations of added sugars, sodium, and saturated fat, so they are a matter for concern [22]. However, they also provide higher concentrations of micronutrients, including shortfall nutrients, such as iron, folate, calcium, and vitamin E [22]. Fortified foods can facilitate the migration to more sustainable diets while meeting nutrient needs [61,62]. Thus, recommendations to eliminate HPFB from the diet pose a tangible risk of compromising diet quality for segments of the population. This risk must be weighed against hypothesized benefits for such an action.

Overall, the epidemiological literature linking changes in the consumption of HPFB to health outcomes does not provide strong evidence of important associations (let alone causal effects) and thus is not a strong foundation on which to make policy.

## Randomized Controlled Trial Data

Though important issues remain to be resolved in the epidemiological literature, that facet of the research effort has amply succeeded in raising an issue of public health concern. Resolution will be aided by evidence from randomized controlled trials. Such data are now becoming available and also warrant a critical review. We are aware of 9 published trials. It would be possible to meta-analyze the trials, but we believe methodological shortcomings (high attrition, short study duration, asymmetrical intervention magnitude, lack of objective compliance measures, poor control of key factors such as food palatability, etc.) in many of the reports, divergent methodologies (community education interventions, community-based dietary manipulation, and metabolic ward studies) and important nuances in the reported findings render such an exercise of little value. Thus, each will be considered individually. A summary of key findings is presented in Table 2.

**TABLE 2:** Summary of randomized controlled trials evaluating ultraprocessed food (UPF) interventions and outcomes

Study	Intervention	Attrition	UPF intake	Appetite	Energy intake	Body weight	Interpretation
Sartorelli et al., 2022 [63]	Educational/clinical (pregnant females)	40% (141/350) for weight outcome	No group difference	—	—	No group difference in gestational weight gain	With no UPF intake difference, no test of the hypothesis
Brandão et al., 2023 [64]	Educational/community (7–12 y/o with OB)	56% (56/101)	No change in UPF intake within or between groups	—	—	Both groups gained weight; control > intervention for BMI and BW. No difference for WC	With no UPF intake difference, no test of the hypothesis
Chen et al., 2022 [19]	Educational/clinical (5–18 y/o with OW/OB)	10% (54/60)	No data collected on UPF intake	—	—	Greater weight loss with lower prescribed UPF diet	With no measure of UPF intake, no test of the hypothesis
Lopes et al., 2025 [65]	Educational/community (adults—range of UPF intake)	26% (2538/3414/)	No group difference in baseline UPF intake; UPF changes not reported	—	—	Weight gain positively associated with UPF intake, but no between-group difference in weight gain for 1st, 2nd or 3rd quartiles of UPF intake. For 4th quartile, greater weight gain with lower UPF intake	With no measure of UPF intake, no test of the hypothesis
Hall et al., 2019 [66]	Metabolic trial (adults—wt stable)	0 (20/20)	%UPF energy 81.3% on UPF and 0% on NUPF	No difference between arms	Increased, but likely transiently	Increased more on the UPF arm, but was inconsistent with energy balance calculations	Effects likely transient
Hamano et al., 2024 [67]	Metabolic trial (adult male with OW/OB)	0 (9/9)	%UPF energy ∼100% on UPF and ∼0% on NUPF	No difference between arms	Increased inconsistently and likely transiently	Both groups gained weight, but increased more during the UPF arm. Shifts in fluid balance cannot be excluded	Effects likely transient
Preston et al., 2025 [68]	Metabolic trial (adult males)	14% (6/43)	%UPF energy 77.1% on UPF and 0.6% on NUPF	—	No difference between groups	No increase with UPF, decrease with MPF	Asymmetrical (no weight gain on high UPF, only weight loss on MPF)
Dickens et al., 2025 [69]	Community-based (adults with OW/OB)	22% (12/55)	Ratio of %UPFen/%NUPFen for each arm:69.2/21.0 and 65.6/23.8	No difference between arms	Reduced in both arms, but lower for NUPF	Decrease on both UPF and NUPF diets, greater loss on the NUPF diet	Asymmetrical (weight loss on high UPF, greater weight loss on MPF)
Macena et al., 2025 [70]	Community-based (adults with OW/OB)	39% (57/148)	%UPF energy 19.96% on UPF and 13.86% on NUPF. No significant group-by-time interaction	No difference between arms	No significant group-by-time interaction	No group-by-time effect for BW, WC, or other body composition indices	No effect
Jardim et al., 2026 [71]	Community-based (children with OW/OB)	44% (73/167)	Intervention group consumed a significantly lower number of UPF foods (%en not reported) (ITT analysis). No significant group difference at end of trial in completers analysis	—	—	No group difference for BMI or BMI/age *z*-score for ITT and completers analyses.	No effect

This table summarizes 9 published randomized controlled trials that aimed to reduce or manipulate ultraprocessed food (UPF) intake using educational/clinical, community-based, or controlled-feeding approaches. For each study, the intervention type, attrition, and reported between-group findings for UPF intake, appetite, energy intake, and body weight are shown, along with a brief interpretation regarding whether the trial meaningfully tests the hypothesized effects of UPF exposure. Attrition is reported as % (and dropouts/total when available). Outcomes summarize between-group findings as reported by study authors; definitions and units vary across trials.

Abbreviations: BW, body weight; ITT, intention-to-treat; MPF, minimally processed food; NUPF, nonultraprocessed food; OW/OB, overweight/obesity; UPF, ultraprocessed food; WC, waist circumference; wt, weight; y/o, years old; %en, percent of energy.

## RCT’s Involving Educational Interventions

Sartorelli et al. [63]—this was a 2-armed, parallel, randomized, controlled trial that assessed the effectiveness of nutrition counseling to replace UPF with MPF (categorized by the NOVA system) coupled with increased physical activity. Participants were 350 females who were pregnant and overweight. The primary outcome was the proportion of females whose weekly gestational weight gain did not exceed the Institute of Medicine guidelines. Attrition was 40%. Although they were not objective measures, there was no significant difference in dietary intake or UPF intake between groups. Body weight data were available from 97 females in the intervention group and 112 females in the control group. No statistically significant difference in gestational weight gain was observed. The failure of the intervention to modify UPF intake limits the interpretability of the trial with respect to the causal role of UPFs in gestational weight gain. In effect, this study does not test the biological or behavioral consequences of reducing UPF consumption, but rather the feasibility of implementing such a dietary change in a real-world prenatal setting. The null result, therefore, likely reflects barriers to adherence and/or limited intervention potency, rather than evidence against an effect of UPF per se.

Brandão et al. [64]—this was a 2-armed, parallel, randomized, controlled, 6-mo trial aimed at reducing UPF consumption to lower energy intake in 101 Brazilian children with obesity aged 7–12 y. Both groups received monthly educational sessions based on the Brazilian Dietary Guidelines; the intervention group additionally received an individualized diet plan designed to promote moderate energy restriction. The primary outcomes were change in BMI, waist circumference, and body weight. Attrition was 56%. There was no difference in UPF intake (not objectively verified) between groups, as both groups showed a transient decrease that returned to baseline. Body weight increased in both groups, with a slightly smaller gain in the intervention group. In contrast, BMI decreased modestly in the intervention group and increased in controls. There was no difference in waist circumference. Because there was no difference in UPF intake, these changes cannot be ascribed to the intervention. Moreover, the modest BMI effect observed in the intervention arm coincided with the only component that differed between groups, explicit caloric prescription, implicating energy restriction rather than food processing as the active element. Interpretation is further complicated by the inclusion of growing children, in whom increases in body weight can coincide with stable or reduced BMI as height increases; age- and sex-standardized indices (e.g., BMI *z*-scores) would have provided a more appropriate metric. Additionally, due to the high attrition rate, the validity of the findings is uncertain.

Chen et al. [19]—this was a 2-armed, parallel, randomized, controlled, 12-wk trial aimed at examining the effects of a Chinese government-recommended diet or one with greater restriction in sugar and UPF, both coupled with increased physical activity, on weight gain. Participants included 60 children and adolescents aged 5–18 y with overweight or obesity. The primary outcome was the change in body weight. Greater weight loss was reported in the group assigned to the more restrictive diet. However, no data were collected on changes in physical activity or actual UPF intake, precluding attribution of the outcome to UPF consumption. The wide developmental range of participants (early childhood through late adolescence) introduces biological and behavioral heterogeneity that is not addressed analytically.

Lopes et al. [65]—this was a 12-mo follow-up of a 2-armed, parallel, randomized, controlled, community trial of 3414 health promotion service users >20 y of age (mean = 56.7y/o). The intervention group received 7 mo of nutrition advice. Body weight was a primary outcome. Attrition was 26%. There was no difference in baseline UPF ingestion or change in body weight over the intervention between the groups. Analyses of body weight were conducted on data with very high levels of imputation for missing data. Dietary intake was only measured at baseline; thus, it is not possible to assess the contribution of UPF intake to body weight changes.

Jardim et al. [71]—this was a 9-mo, randomized, parallel-group, community education-based trial involving 167 Brazilian children aged 6–10 y with BMI *z*-scores ≥+2. The aim was to evaluate the effect of a dietary intervention involving a reduction of UPF consumption on the nutritional status of children receiving primary health care services. Ninety-four children completed the trial, resulting in an attrition of 44%. The intervention group received ≥26 activity contact hours, whereas the control group was provided only 9. UPF intake was assessed as the number of such foods consumed based on a 36-item food frequency questionnaire administered for 1 d on 5 occasions. Neither energy intake nor energy expenditure was measured. There was no objective measure of dietary intake. The number of UPFs consumed was significantly reduced in the intervention group compared with the control group (difference ∼1 item/d). There was no group difference for BMI or BMI/age *z*-score. The lack of a significant between-group difference in UPF intake at the end of the trial by study completers suggests a reduction of adherence over time.

Overall, the trials implementing an educational intervention have assessed a good representation of the population age distribution, including pregnant females, children, adolescents, and adults. However, they have provided limited new insights on the contribution of UPF intake on weight management because 2 studies [63,64] did not achieve a differential UPF intake in the study arms, and 2 others [19,65] did not measure UPF intake during the trial. Additionally, 4 [19,63,64,71] had high attrition rates, raising concerns about self-selection bias. Three trials reported no between-group differences in weight change; one observed weight gain in both groups, though less for the group more restricted in UPF intake, and one noted a greater loss of body weight, so there is no reliable effect. Perhaps the clearest message to be gleaned from this work is that implementation of a lower UPF diet may be difficult to achieve.

## RCT’s Involving Food Interventions

### Clinical ward setting

Hall et al. [66]—this was a randomized, controlled, crossover design study conducted in a metabolic ward with 20 weight-stable adults (mean BMI 27kg/m^2^; mean age 31.2 y). UPF contributed 81.3% en on the high UPF diet and 0% en on the MPF diet, so this was a proof-of-principle trial rather than a test of intake level practiced in any population [72,73]. The diets were administered as free feeds for 2 wk each with no washout between. The primary outcome was mean ad libitum energy intake over each 14-d diet period. Energy intake and body weight were greater during the UPF arm compared with the MPF arm. Energy intake did not differ significantly during the MPF arm, but there was a significant linear decrease of 25.5 kcal/d during the UPF arm, indicating adaptation. Extrapolation of this linear trend indicates that the between-diet difference would have approached zero within 2 additional weeks. Such a transient effect is of questionable nutritional importance. Energy intake was greater at breakfast and lunch meals, but not at dinner or snacking, suggesting meal-specific effects not attributable to UPF. Additionally, short-term energy balance calculations were inconsistent with the observed weight changes (i.e., intake minus expenditure was slightly positive during the MPF arm and there was no decrease in energy intake over that phase despite weight loss), which may reflect contributions from water, glycogen, and gut content rather than fat mass alone. The sample size was reasonable for a metabolic ward trial, but very small for extrapolating findings to the greater population, let alone translation of ingestive behaviors from such a setting to free-living individuals. Participants did not report differences in the sensory appeal of the foods or appetitive sensations between the diets. Meal eating rate differed, but other work has demonstrated that food form, rather than UPF status, is the dominant driver of eating rate (Teo et. al., 2022), and the UPF and MPF diets differed markedly in physical form, as well as added sugar content and fiber source. Overall, each dietary phase lasted only 2 wk, a duration sufficient to capture acute behavioral responses but not possible longer-term behavioral or biological compensation. Additionally, participants resided in a metabolic ward and did not shop, choose foods, face cost constraints, engage in social eating, or experience advertising or availability pressures. Thus, the environmental features hypothesized to render UPFs problematic were removed.

Hamano et al. [67]—this was a randomized, controlled, crossover design study conducted in a hospital with 9 males with overweight/obesity (mean age 29.7 y; mean BMI = 27.4 kg/m^2^). Participants consumed diets entirely of either low (0% en) or high (100% en) UPF, as defined by NOVA classification, for 1 wk each, with a 2-wk washout between. Thus, the diets were more extreme than any consumed by a free-living population [72,73]. Although framed as a test of “processing,” the diets differed on multiple dimensions beyond degree of processing, including fiber content, salt, water, texture, and physical form. The primary outcome was the difference in the body weight change between arms. Participants consumed more energy and gained more weight on the UPF arm. However, no data on trends in energy intake were reported, and the data presented indicate the differences varied across days and were trivial by the end of the week. This, again, suggests a transient effect. As with the Hall study [66], excess energy intake was observed only at selected eating occasions; here, at lunch and dinner, but not breakfast or snacks, once more suggesting a meal-specific effect not attributable to UPF. Extrapolation of findings from a small sample, here only 9 males over a 1-wk exposure, with weight as the primary outcome, is questionable. The intake of salt and water was greater on the UPF arm raising the possibility that the changes in body weight reflected shifts in fluid balance.

### Community setting

Preston et al. [68]—this was a randomized, controlled, crossover design study conducted with 43, free-living, adult males (mean age ∼26 y; mean BMI ∼23.5 kg/m^2^). Participants consumed diets extremely low (∼0.6% en) or high (∼77.1% en) in UPF for 3 wk with a 12-wk washout between treatment arms. Participants were additionally stratified into an “adequate calories” compared with “excess calories” condition, with analyzed sample sizes of *n* = 20/18 and *n* = 21/20 across diet sequences and caloric arms. The test foods were provided to the free-living participants, but there was no objective measure of dietary compliance. Habitual UPF intake at baseline was ∼51% of energy, whereas the intervention diets provided 77% and 1% energy from UPFs in the UPF and nonultraprocessed food (NUPF) arms, respectively, rendering the intervention magnitude asymmetrical, especially large for the MPF arm. The diets were more extreme than reported in any population [72,73] and were composed of different foods that varied not just by degree of processing or formulation. The primary outcome was whether UPF impairs reproductive and metabolic fitness, with further aggravation by excess caloric intake. There was no significant difference in energy intake between the UPF and MPF arms in either caloric condition. Body weight differed between UPF and MPF arms, but due to a reduction on the MPF diet. There was no significant weight change on the UPF diet. The reduction of body weight only in the MPF arm, despite no differences in energy intake, raises questions about the validity of the intake data or a plausible mechanism.

Dicken et al. [69]—this was a randomized crossover, controlled-feeding trial involving 55 adults (mean = 43.2 y; mean BMI = 32.72 kg/m^2^). Participants were provided a UPF diet as defined by NOVA classification or MPF variation of the UK Eatwell Guide diet for 2 8-wk free-feeding periods with a 4-wk washout between. All foods were delivered to the participant’s homes. The primary outcome was the within-participant difference in percent weight change between diets, from baseline to week 8. Mean baseline UPF intake was 67.3% en. The MPF intervention provided 1.7% en from UPF and the UPF diet provided 90.7% en from UPF, again rendering the magnitude of intervention asymmetrical and greater on the MPF arm as well as more extreme than any reported population [72,73]. Both groups lost weight with greater loss on the MPF arm. However, there was a significant order effect: the between-diet difference in weight occurred only during the first treatment arm. Ten participants withdrew from the trial during the MPF arm and 2 more during the washout, whereas none withdrew during the UPF arm raising questions about possible self-selection bias. The self-reported sensory appeal of the diets was lower on the MPF arm. This may have contributed to the selective attrition or difficulty adhering to the dietary intervention over time. Self-reported diet adherence was lower during the second arm. There was no treatment effect on appetite.

Macena et al. [70]—this was a 12 mo, 2 parallel-arm randomized clinical trial. The primary aim was to evaluate the effectiveness and metabolic effects of restricting UPF consumption in individuals with obesity undergoing energy restriction. One hundred forty-eight participants were enrolled, but only 91 completed the trial; an attrition rate of 39%. Baseline UPF intake was 21.16% of energy in the UPF-restricted group and 20.02% en in the control energy-restricted group. The intervention targeted ≤5% of energy from UPFs, but the achieved intake in the UPF-restricted group was ∼14% energy at 12 mo; consistent with other trials experiencing difficulty in achieving marked reduction of UPF intake. The control group also reduced UPF intake slightly (≈20%), so there was no group-by-time interaction. A small statistical advantage in weight loss emerged when monthly measurements were modeled (*P* = 0.01), but the magnitude was clinically trivial (≈2% loss in the intervention group compared with ≈1% in controls), with no between-group differences in waist circumference, body composition, energy expenditure, or biochemical markers. No differences were observed for any of the measured appetitive sensations.

Taken together, based on methodological issues and the reported outcomes, findings from food intervention trials provide little support for the hypothesis that ingestion of processed food is problematic for weight management. Consumption of a high UPF diet led to increased weight [66,67], no change in weight [68,70] and weight loss [69]. The 2 trials conducted in metabolic wards were interventions with 9 males for 1 wk [67] or 20 adults for 2 wk [66]. Both showed no group differences of appetite, only transient effects on food intake and equivocal changes of weight [i.e., were not consistent with intake data (Hall) or were subject to alternate interpretations] [67]. Furthermore, both used diets more extreme in UPF and MPF foods than those observed in any free-living population [72,73]. Thus, neither was of a size, duration, or ecologically valid intervention that would permit extrapolation of findings to the general population. Three trials were community based. None included an objective measure of dietary compliance and 2 had high attrition rates [69,70]. One administered an ecologically questionable difference in UPF intake [68] and another was not able to achieve the intended intervention [70]. Two of the trials reported no group differences for appetite [69,70] and 2 [69,70] reported no group-by-time interaction for energy intake [68,70]. None observed weight gain on the high UPF diet and only 2 noted a reduction of weight on the MPF diet [68,69], due possibly to the greater deviation from customary intake on that arm and lower palatability of the MPF foods [69]. One of those studies had an order effect such that the change in weight was only apparent on the first arm of the crossover [69]. To establish whether any of the proposed food classification systems provides insights on causal relationships between food processing and health will require the conduct of longer-term clinical trials with larger study samples, ecologically plausible interventions, improved approaches to implement dietary changes, and strong compliance measures.

### Mechanisms

Numerous mechanisms have been hypothesized to underlie the effects of processed food intake on body weight. However, an evidence base supported by empirical data remains lacking [74]. Indeed, evidence challenging some of the most commonly cited mechanisms is accumulating. For example, the hypothesis that processing renders foods less satiating is now refuted by multiple RCTs [55,58,60,61]. Claims that UPF foods are problematic for weight gain because they are rapidly consumed have been shown to be attributable to food form more than processing classification [[75], [76], [77]]. Additionally, the argument that processed foods are hyperpalatable is not supported by RCTs that reported no difference in sensory appeal of UPF and NUPF diets [55], no change of intake (the presumed result of greater palatability) [59] and, in 1 case [60], a reduction of body weight suggestive of reduced intake. This latter purported mechanism is mistakenly based on a view that palatability is a property of a food when common experience shows the appeal of any food is determined by individual characteristics such as culture, age, genetics, and health. In other cases, adverse effects of processed foods on biological systems, such as the microbiome [78,79], continue to be claimed while acknowledging no mechanism has been identified.

Despite the absence of a clear definition for the concept, weak effects documented by observational findings, and inconsistent findings from randomized controlled trials (all using the NOVA classification system), it is vital that understanding of how food processing and formulation influence health be expanded. To meet the nutritional needs of the present population, let alone a growing population [80] and assure the food system meets expectations for food availability, accessibility, affordability, and desirability [81], processing of food is and will be required. It has been argued that processing has enhanced diet quantity and quality, food safety (overall and for individuals with specific food allergies/sensitivities), as well as convenience and reduced food waste [82], whereas others contend processing has had deleterious effects [[82], [83], [84], [85]]. The reality is that there is validity to both perspectives so the challenge is to critically assess current practices and processes to determine their net effect and to explore ways to augment the benefits and minimize the unintended, undesirable consequences [27,82,86,87]. A set of guiding principles for undertaking such work has recently been published [88]. Until progress is made in this regard, the tangible health risks and ethical concerns with eliminating HPFB from the diet and the lack of compelling evidence that reduction or elimination of foods classified as HPFB will be beneficial, it is premature and likely harmful to base dietary guidance on such a concept.

In conclusion, there is little doubt that alteration of the physical properties of foods and their composition holds implications for the likely physiological and health consequences of their consumption. However, claims that processing is a prepotent factor is less clear. There is abundant observational evidence linking food classified as highly processed with a wide array of health disorders, but the effect sizes are small, inconsistencies in such findings are notable, and the quality of the data is consistently rated as very weak or weak. Moreover, the diet quality of some segments of the population is improved by consumption of foods labeled as problematic based on processing alone. Evidence from a small but growing body of randomized controlled trials, where questions about causality may be addressed, reveal a spectrum of outcomes ranging from no effect to augmented energy intake (possibly only transiently), and decreased, no change, or increased body weight. The trials typically had small samples, high attrition, and poor success in implementing the planned intervention. The latest, largest, longest, and most ecologically valid trial revealed no meaningful effect on body weight [61]. There is also a lack of evidence supporting any proposed mechanism. Taken together, current evidence does not conclusively establish food processing, independent of other dietary characteristics, as a major causal determinant of obesity risk. Given the important contributions of food processing to food safety, preservation, affordability, convenience, and nutrient adequacy, caution is warranted before translating current evidence into broad dietary guidance or regulatory policy.

## Author contributions

The authors’ responsibilities were as follows—RDM: conceived the review, wrote the first draft, and holds primary responsibility for the final content; GR: conducted the statistical analyses and edited the draft; JB: edited the draft; and all authors: read and approved the final manuscript.

## Data availability

Data described in the manuscript and the R code will not be made available because the UPFB sales inputs were digitized from a copyrighted published figure and are subject to licensing/copyright restrictions that prevent public redistribution.

## Declaration of Generative AI and AI-assisted technologies in the writing process

The authors declare that no generative AI or AI-assisted technologies were used in the writing of this manuscript.

## Funding

This study was supported by the Institutional Funds.

## Conflict of interest

The authors report no conflicts of interest.
